# Promoting planting in front gardens: a systematic approach to intervention development

**DOI:** 10.14324/111.444/ucloe.3147

**Published:** 2024-05-24

**Authors:** Ayşe Lisa Allison, Rachael Frost, Niamh Murtagh

**Affiliations:** 1 UCL Centre for Behaviour Change, University College London (UCL), 1-19 Torrington Pl, London WC1E 7HB, UK; 2 UCL Plastic Waste Innovation Hub, University College London (UCL), 90 Tottenham Court Road, London W1T 4TJ, UK; 3 Department of Primary Care and Public Health, University College London (UCL), UCL Royal Free Campus, Rowland Hill Street, London NW3 2PF, UK; 4 The Bartlett School of Sustainable Construction, University College London (UCL), 1-19 Torrington Pl, London WC1E 7HB, UK

**Keywords:** Behaviour Change Wheel, biodiversity conservation, front gardens, gardening, public engagement, public health, sustainability

## Abstract

Planting in front gardens is associated with a range of human and environmental health benefits. Effective interventions aimed at cultivating this practice are, however, hampered by the paucity of theory- and evidence-based behavioural research in this context. This study aims to systematically determine a set of behaviour change interventions likely to be effective at promoting planting in front gardens amongst UK householders. The Behaviour Change Wheel framework was applied. Behavioural systems mapping was used to identify community actors relevant to front gardening. Potential behavioural influences on householders’ front gardening were identified using the Capability, Opportunity, Motivation, Behaviour model. Using peer-reviewed scientific findings as evidence, behavioural influences were systematically linked to potential intervention strategies, behaviour change techniques and real-world implementation options. Finally, intervention recommendations were refined through expert evaluations and local councillor and public stakeholder feedback, evaluating them against the Acceptability, Practicability, Effectiveness, Affordability, Side effects and Equity criteria in a UK implementation context. This study formulated 12 intervention recommendations, implementable at a community level, to promote front gardening. Stakeholder feedback revealed a preference for educational and supportive (social and practical) strategies (e.g., community gardening workshops, front gardening ‘starter kits’) over persuasive and motivational approaches (e.g., social marketing, motivational letters from the council to householders). Householders’ front gardening behaviour is complex and influenced by the behaviour of many other community actors. It also needs to be understood as a step in a continuum of other behaviours (e.g., clearing land, gardening, waste disposal). This study demonstrates the application of behavioural science to an understudied implementation context, that is, front gardening promotion, drawing on a rigorous development process promoting a transparent approach to intervention design. Stakeholder consultation allowed relevance, feasibility and practical issues to be considered. These improve the likely effectiveness of interventions in practice. The next steps include evaluating the proposed interventions in practice.

## Introduction

Gardening offers a range of physical, psychological and social health benefits to humans [1,2] and contributes towards biodiversity conservation [3]. This is particularly so in urban environments where green space (e.g., parks, grasslands) is often on the decline [4]. The physical activity involved in gardening, such as digging, planting, weeding and watering, can contribute to regular exercise, which is essential for health [5]. There are also a range of mental health benefits. Gardening can reduce stress and anxiety by providing a calming environment, a sense of meaning and a connection to nature, thereby promoting mental well-being [6]. In this study, we focus specifically on gardening in front gardens. For the scope of this study, front gardens are defined broadly as the communally or privately owned or rented space between the front of the dwelling and the street that is accessible to householders and large enough for three recycling bins (i.e., at least 3 m^2^).

Front gardening, in particular, has additional benefits to gardening in back gardens which are often private and secluded from view – it can serve as a focal point for social cohesion through providing social interaction with neighbours and the wider community [7]. The opportunities for social interaction provided by front gardens can foster a sense of belonging and reduce feelings of isolation, supporting social factors imperative for the long-term health and well-being of citizens [8]. Aside from the social benefits offered by front gardens, growing plants in front gardens can also help to regulate extremes in temperature during heat waves, provide shelter and insulation in winter [9,10] and reduce the risk of urban flooding [11]. While the specific benefits derived from growing in front gardens can depend on the size of the garden, the types of plants cultivated (e.g., the benefits of homegrown produce from edible plants such as fruits, herbs and vegetables) and the overall level of maintenance involved, the evidence strongly shows that cultivating a front garden can contribute to a healthier and more meaningful lifestyle for people [7,12–14].

It is a growing public and environmental health concern then that over five million front gardens (about a third) in the UK now have no plants growing in them, and four and a half million front gardens (one in four) are completely paved over [15]. Reasons for this include increasing fees and regulations for road parking, a desire for lower garden maintenance requirements and a lack of time or skills to look after green space [16]. The health and environmental consequences of paving over a front garden remain largely unknown, though evidence from the UK suggests that it can increase the risk of flooding [11,17] and is likely to reduce the psychologically restorative and community-building benefits of visible front garden greenery [7]. The Royal Horticultural Society, the main gardening charity in the UK, has long been campaigning and funding research to protect front gardens [15,18]. Due to the social, psychological and environmental health benefits provided by front gardens, there is a growing research interest in understanding and cultivating this practice amongst UK citizens [7,12–14,19–21].

For instance, aside from Chalmin-Pui et al. [12], few interventions have been developed and evaluated to promote front gardening amongst UK citizens. Chalmin-Pui et al.’s [12] intervention consisted of introducing ornamental plants to 38 previously bare front gardens (≈10 m^2^) within an economically deprived region of Northern England. The findings showed significant decreases in perceived stress post-intervention, which aligned with a higher proportion of ‘healthy’ diurnal cortisol patterns. Qualitative results corroborated these findings by showing that residents valued their front gardens as they enhanced relaxation, and increased positive emotions, motivation and pride of place. Just adding small quantities of ornamental plants to front gardens had a positive effect on individuals’ stress regulation and some (though not all) aspects of subjective well-being in the community where the intervention was implemented. Nonetheless, a limitation of this study is that the rationale for the intervention approach was not clear – it was not developed using behaviour change theory or intervention development frameworks. While the study shows that adding plants and containers to householders’ front gardens leads to positive health and well-being benefits, it does not provide an in-depth exploration of the barriers and enablers to front gardening which is critical to design effective interventions that promote this practice.

While some preliminary research into the barriers and enablers to front gardening has been conducted in the UK [20,21], these findings have yet to be systematically integrated into a comprehensive set of practical intervention recommendations. Frost and Murtagh [21] conducted a qualitative focus group study and found that a desire for health benefits (e.g., fresh air and vitamin D) enabled front gardening. Planting in front gardens also depended heavily on environmental context (e.g., available time and space, garden orientation, local security and the weather). Murtagh and Frost [20] conducted a quantitative survey which showed that reasons for front gardening included enjoyment, meaning, health benefits, creating something beautiful and functional outcomes. The next step is moving from an understanding of the potential influences on front gardening behaviour to concrete intervention strategies that can bring about the desired behaviour change.

### Enabling behaviour change

Promoting front gardening amongst the UK public is complex – it requires people to adopt a new set of behaviours that may be foreign and challenging to them. As a result, effectively and sustainably changing behaviour requires systematic, theory- and evidence-informed approaches to intervention design. The scarcity of research focussed on intervention development in this area does not allow for the leveraging of identified influences on front gardening and the overcoming of barriers to promote this practice. Interventions can be implemented that have face validity but miss important influences that drive behaviour or contextual and implementation factors and therefore may not be as effective as they potentially could be. This is exemplified by Kelly and Barker, who highlight key errors policymakers make when trying to change public health-related behaviours [22]. Examples of the decision-making errors identified include assuming that behaviour change is just ‘common sense’, only about getting the message across or that knowledge and information are the key drivers of behaviour. Kelly and Barker maintain that behaviour change requires a careful and considered science sensitive to the various factors that influence people’s behaviours. Contextual factors may be critical, concerning particular groups or cultures, available resources, history of interventions or equity and so require tailoring to context.

Enabling behaviour change is therefore not easy. Research aimed at developing and evaluating the kinds of ‘complex’ interventions needed to achieve behaviour change argues for theoretically grounded and evidence-informed approaches [23–25]. Evidence shows that application of behaviour change theory can improve the development of behaviour change interventions [26,27]. To facilitate this process, a variety of frameworks have been developed, and widely used, to assist the process. In this study, we aim to address the gap on systematically designed interventions within the literature on front gardening by applying behavioural science principles, methods and frameworks, informed by stakeholder consultation, to the promotion of front gardening amongst UK householders.

As there is a paucity of documented intervention efforts in this area, in this study we aim to provide intervention recommendations that could be implementable at a local authority or community level in the first instance. We recognise that wider structural changes to urban planning and housing infrastructure would also likely be valuable to engage more UK householders in this practice. However, we chose to focus the study at a local level, noting that changing national policy is an area for further investigation.

### Theoretical behaviour change frameworks

Widely used and advocated by both local and national UK Governments as a suitable behaviour change tool [28–30] is the Behaviour Change Wheel (BCW) intervention development framework [31,32]. Benefits of the BCW include its provision of a structured approach to designing and evaluating behaviour change interventions, which can include interventions for individuals, organisations and populations. The purpose of the BCW is to provide a systematic and comprehensive analysis of available intervention options for a given behaviour change challenge, to identify those most likely to be effective. The BCW is used frequently in many areas of research, most frequently to health, for example, patient and healthcare provider behaviour change [33–37], but has more recently seen its expansion into sustainability behaviour change research [38,39]. To the best of our knowledge, it has had no application within the context of promoting gardening. Given the range of societal benefits promised by front gardens, there is value in exploring the BCW’s application to intervention design within this area and identifying behaviour change recommendations.

**Figure 1:**
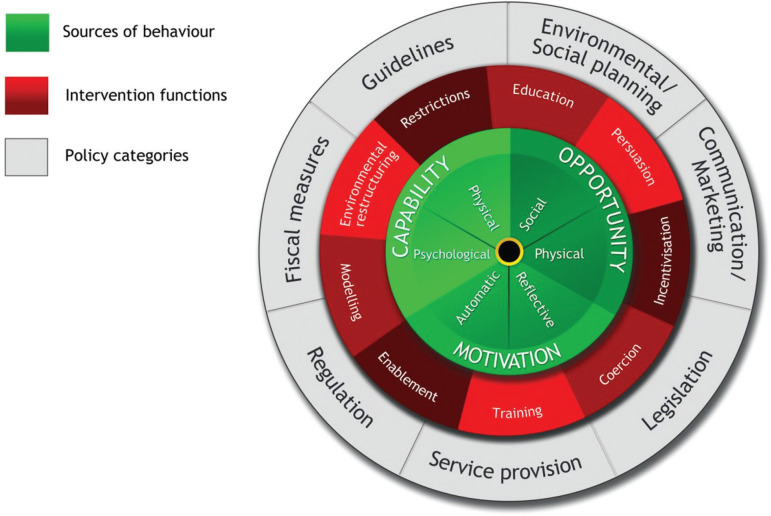
The BCW [31,32].

Shown in Fig. 1, the BCW defines a process of intervention design starting from the inner hub of the wheel and working outwards. The wheel itself consists of three parts: 1) an inner hub which represents what needs to be targeted to achieve the desired behaviour change in terms of capability, opportunity and/or motivation; 2) a middle layer of ‘intervention types’ which represent broad categories of how to change behaviour; and 3) an outer layer which are policy options for delivering the intervention. Definitions of each intervention type and policy option can be found in Table 1. As noted above, selecting potential policy options were deemed outside the scope of the present study and so in this study this stage in the BCW process was skipped.

**Table 1.** Table showing definitions of BCW intervention types

Intervention type	Definition
Education	Increasing knowledge and understanding
Persuasion	Getting people to change behaviour by generating ‘cognitive dissonance’ – an uncomfortable state of having contradictory beliefs, thoughts or values towards something [40]
Incentivisation	Changing the attractiveness of a behaviour by creating the expectation of reward
Coercion	Changing the attractiveness of a behaviour by creating the expectation of punishment
Training	Increasing psychological or physical skills
Restriction	Constraining behaviour by setting boundaries
Environmental restructuring	Altering the physical or social environment
Modelling	Showing examples of the behaviour for people to imitate
Enablement	Providing support to change behaviour in ways not covered by other intervention functions, for example, through encouragement, moral support
Policy options	
Guidelines	Development and dissemination of documents that make recommendations for desired behaviour
Environmental and social planning	Changing the physical and social environment people inhabit
Communications and marketing	Use of marketing channels and tools to communicate a message, for example, can include mass media campaigns and digital marketing campaigns
Legislation	Using laws and other similar instruments to set the restrictions on behaviour with penalties for breaching
Service provision	Providing a service, material resource and aids
Regulation	Development and implementation of rules regarding behaviour that instruct the behaviour and possibly provide rewards and punishments for conforming
Fiscal measures	Use of taxation and tax relief. The aim here is to incentivise and disincentivise behaviours where one has authority to levy taxes

In terms of methodology, the BCW advocates three broad stages: 1) understanding the target behaviour in terms of people’s capability, opportunity and motivation, 2) selecting the most appropriate intervention types (and policy options, if relevant to your context) based on the evidence and, 3) selecting content and implementation options in terms of specific behaviour change techniques (BCTs) and modes of delivering the interventions in practice.

There are ancillary methods and frameworks as part of the wider BCW process which facilitate progressing through these three broad steps. These include behavioural systems mapping, the Capability, Opportunity, Motivation, Behaviour (COM-B model) [32], the BCTs taxonomy [41] and the Acceptability, Practicability, Effectiveness, Affordability, Side effects and Equity (APEASE) framework [31].

#### Behavioural systems mapping

What might seem like a simple behaviour is often highly complex and influenced by the behaviours of other people. Behavioural systems mapping is an emerging methodology that can used to effectively identify and understand actors within a behavioural system (e.g., broad groups of people, their actions and behavioural influences) and map out the relationships between these entities. The core idea behind behavioural systems mapping is to provide a holistic view of a system’s dynamics by visually representing how it is upheld by the relationships between people and their actions.

These maps can help with decision-making, problem-solving and system optimisation. They are useful starting points to conceptualise complex problems (such as urban biodiversity conservation or community health and well-being) in behavioural terms. They can also help to identify ‘entry points’ for interventions, for instance, by illustrating the broad groups of people who could potentially implement a behaviour change intervention. They can also be used to help identify other behaviours that might need to also be changed in order to bring about a change in a desired target behaviour. Readers are referred to Hale et al. for an example of a behavioural system mapping approach, linked to the BCW framework, to develop policy recommendations with population-level behaviour change as the primary objective [42].

#### COM-B model

**Figure 2:**
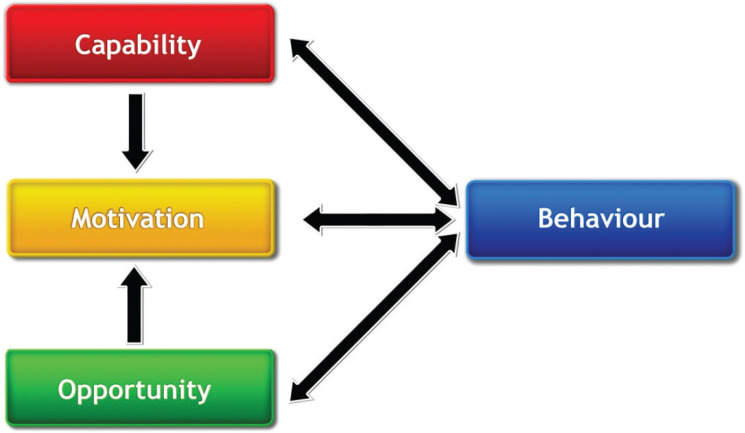
The COM-B model [31,32].

The COM-B model (Fig. 2) is at the hub of the BCW and offers valuable support for identifying what needs to change to bring about desired behaviour change. COM-B posits that there must be Capability, Opportunity and Motivation for behaviour to occur. Capability refers to people’s physical or psychological capability, such as their physique and stamina or knowledge, intellectual capacity and memory and decision-making processes. Opportunity refers to social or physical opportunity such as the social environment of cultures and norms or the physical environment of objects and events with which people interact. Motivation can be automatic or reflective motivation and refers to the intentions, desires, evaluations, habits and instincts that direct human behaviour.

#### The BCTs taxonomy

The taxonomy of BCTs is a taxonomy comprising 93 hierarchically clustered BCTs [41] and ways of implementing the BCTs in practice. BCTs can be thought of as the elementary components of interventions such as ‘goal setting’, ‘action planning’ or ‘instructions on how to perform the behaviour’. Definitions of each BCT can be found in the original paper [41].

#### APEASE

As behaviour change interventions occur in ‘real world’ social, economic and political contexts, these types of contextual factors must be taken into consideration during the design process to maximise the likely effectiveness and success of implementation efforts. As part of the BCW set of resources, the APEASE framework is provided to structure this process ([31], see Table 2), The overall purpose of APEASE is to enhance the likelihood of relevance, utility, equity and practicability of an intervention, to support the selection of promising interventions, or the refinement of potentially ‘problematic’ interventions.

**Table 2.** APEASE criteria definitions

APEASE criteria	Definitions
Acceptability	How appropriate the intervention is deemed by key stakeholders and those targeted by the intervention
Practicability	How practically feasible the intervention will be in the intended setting
Effectiveness	How effective the intervention will be at changing the target behaviour
Affordability	How costly the proposed intervention will be
Side effects	A consideration of potential unwanted side effects from the intervention
Equity	A consideration of whether the intervention reinforces disparities between different sectors of society

### The present study

The primary aim of this study is to determine an appropriate set of behaviour change intervention recommendations that promote front gardening amongst UK householders. A secondary aim is to develop these recommendations via systematically applying a behaviour change intervention development framework – the Behaviour Change Wheel.

## Method

**Figure 3:**
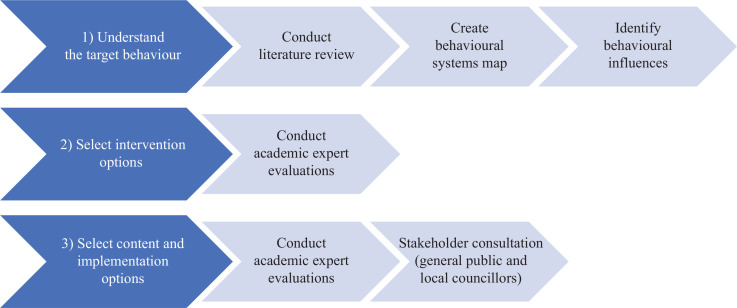
Summary of the intervention recommendation development process informed by BCW guidance.

Applying the BCW and its ancillary frameworks, the process followed to determine behaviour change intervention recommendations is summarised in Fig. 3.

### Understand the target behaviour

#### Conduct literature review

Key papers were identified by each author, supplemented with rapid literature searches. Given the paucity of empirical evidence in this area, a decision to include non-UK studies was made. This also allowed for a wider range of potential contextual factors to be considered.

Papers that investigated influences on front gardening and/or evaluated interventions aimed at changing front gardening behaviour were included. Papers were excluded if they did not specifically refer to front gardens. This is because, in the UK, planting in front gardens is behaviourally distinct from back gardens (which are often private and secluded places), as such the barriers and enablers were likely to be different. The review identified seven papers [7,12,14,20,21,43,44] (see Table 3).

**Table 3.** Characteristics of studies identified from the literature review

Paper	Author and year	Country	Population	Methods	Focus
[43]	Hunter and Brown, 2012	USA	All private properties within Ann Arbor (n = 22,562) with gardens (n = 2562)	Spatial clustering analysis	Social contagion effects of growing in front gardens
[44]	Afrad and Kawazoe, 2020	Morocco	Residents of densely populated, disadvantaged neighbourhood, Beni-Makada district of Tangier, Morocco (n = 388)	Face-to-face survey, ordinary least squared regression analysis	Investigate the association between ownership of a potted street garden and depression levels
[14]	Chalmin-Pui et al., 2021	UK	n = 6015 members of UK general population	Quantitative survey; regression analyses	Attitudes and perceived health benefits of home gardening
[12]	Chalmin-Pui et al., 2021	UK	n = 42 residents from Salford, Manchester	Pre/post measurements of perceived stress and diurnal cortisol profiles (as an indicator of health status); qualitative evaluation of intervention perceived benefits	Front garden growing intervention evaluation
[7]	Chalmin-Pui et al., 2023	UK	n = 20 Britain in Bloom gardeners in Greater London	Focus group study, interpretive phenomenological analysis	Gardening motivations and health and social cohesion impacts for gardeners, residents and passersby
[20]	Murtagh and Frost, 2023	UK	n = 1000 members of UK general population	Quantitative survey; regression analyses; COM-B model used as theoretical framework	Influences on growing in front garden in the UK
[21]	Frost and Murtagh, 2023	UK	n = 20 residents aged 20–64 in England	Focus group study; thematic analysis; COM-B model used as theoretical framework	Influences on growing in front garden in the UK

#### Create behavioural systems map

A behavioural systems map of the key actors (i.e., broad groups of people), their behaviours and the relationships between these entities, was created to visualise the system relating to UK householders’ front gardening. The map was developed based on the literature review and drawing on the authors’ previous research in the area. The data visualisation software Kumu (https://kumu.io/) supported this process.

#### Identifying behavioural influences

Potential influences on front gardening behaviour were identified from the literature review. Identified behavioural influences were initially categorised according to COM-B (i.e., physical capability, psychological capability, social opportunity, physical opportunity, automatic motivation and reflective motivation) by the lead author (ALA). The findings were then reviewed by the co-authors (RF and NM) to achieve a consensus on how influences were categorised into COM-B. No major discrepancies were identified and minor discrepancies were discussed until resolved. Table 4 narratively summarises these findings in terms of barriers and enablers to front gardening.

**Table 4.** Summary of COM-B behavioural influence findings from the literature review

COM-B factor	Summary of findings
Physical capability (n = 2)	Issues with physical pain and mobility were barriers based on the physical exertion required (−) while others viewed gardening as an inclusive practice that could be adapted to meet the needs of each person’s physical skill, stamina and mobility (+) [7,14].
Psychological capability (n = 3)	Enablers included an awareness of the community and environmental health benefits of growing in front gardens and having the necessary procedural knowledge of how to garden for one’s own gardening conditions (e.g., having a good knowledge of plants including terminology, what can grow where and under what conditions and how to care for them) (+) while barriers included issues with mental health, for example, anxiety and depression (−) [7,14,21].
Social opportunity (n = 6)	Low sense of community including a high perceived threat of vandalism and theft was a deterrent to front gardening (−) [20,21,44]. Neighbourhood norms and social contagion as a mechanism of behavioural change, for example, clustering of visually appealing gardens enabled front gardening (+) [12,43]. Prior personal experience of gardening was also an enabler of gardening in front gardens – this was usually in the form of having learnt from previous generations (+) [7,14,20,21].
Physical opportunity (n = 4)	Not having the time, funds or appropriate space, garden conditions or location to grow were barriers (−) whereas having these things were enablers (+) [12,14,20,21]. Inheriting plants from previous homeowners or tenants made householders more likely to maintain them (+) [21].
Automatic motivation (n = 4)	This manifested as the rewarding feelings associated with gardening, for example, enjoyment and relaxation (+) [7,12,14,21].
Reflective motivation (n = 6)	Enablers included having a high sense of self-efficacy, confidence in one’s gardening capabilities, growing in front gardens as a form of creativity and self-expression and alignment of the behaviour with self-identify, for example, deriving a sense of pride, meaning, responsibility and connectedness from it (+) [7,12,14,20,21,44]. Barriers related to an absence of motivations to front garden. Some residents had more pressing prioritie, for example, renters who do not wish to invest more resources for a home they do not own or residents preferring to prioritise their back gardens vs their front gardens (−) [20]. Distinct from the physical opportunity related external constraints on time and cost were also reflective motivation related perceptions of constraints on time and cost. In these instances, these factors were more indicative of motivational and priority-related barriers to front gardening (−) [20].

Notes: n represents the number of studies where each COM-B factor appears.

+/− distinguish between barriers (−) and enablers (+) to front gardening.

### Select intervention options

#### Conduct academic expert evaluations

Based on BCW guidance [31], the intervention types most likely to be effective were selected to target the identified COM-B influences. The potentially relevant intervention types to support delivery of the interventions were also evaluated against the APEASE criteria to decide whether or not they should be moved forward to the next stage of intervention design. The selection of intervention types and APEASE evaluations were initially conducted by the lead author (ALA) and independently reviewed by the other co-authors (RF and NM) to achieve consensus. No major discrepancies were noted and minor discrepancies were discussed until resolved. The academic expert evaluations for the intervention types are shown in Appendix A.

### Identify content and implementation options

#### Conduct academic expert evaluations

Drawing on the BCT taxonomy [41] introduced alrady, potential BCTs were identified by the lead author (ALA), and independently reviewed by the other co-authors (RF and NM) (Appendix B). The final 19 BCTs selected are shown in Table 5. To remind the reader, the definitions of each BCW intervention type is reiterated in Table 5. Concerning COM-B, psychological capability refers to aspects such as knowledge, intellectual capacity, memory and decision-making processes while physical capability refers to aspects such as physique and stamina. Physical opportunity refers to the physical environment of objects and events with which people interact while social opportunity refers to the social environment of cultures and norms. Reflective motivation refers to the conscious intentions, desires and evaluations that direct behaviour while automatic motivation refers to the unconscious habits, feelings and instincts that direct behaviour.

**Table 5.** Selected BCTs from the academic expert evaluations

BCW intervention type selected	COM-B component targeted	BCTs selected to target key behavioural influences identified
Education (Increasing knowledge and understanding)	Psychological capability, that is, knowledge of front gardening benefits, plant knowledge	Information about social and environmental consequences Information about health consequences Information about emotional consequences
Persuasion (Getting people to change behaviour by generating ‘cognitive dissonance’ – an uncomfortable state of having contradictory beliefs, thoughts or values towards something)	Automatic motivation, that is, rewarding feelings associated with gardening, for example, enjoyment and relaxation Reflective motivation, that is, gardening as form of creativity/self-expression and identity, for example, pride, connectedness, responsibility, civic duty. High self-efficacy and confidence in capabilities	Information about social and environmental consequences Information about health consequences Focus on past success Verbal persuasion about capability Identity association with changed behaviour Identification of self as role model Information about emotional consequences Information about others’ approval Social comparison
Incentivisation (Changing the attractiveness of a behaviour by creating the expectation of reward)	Reflective motivation, that is, competing priorities and absence of motivation to front garden	Incentive
Training (Increasing psychological or physical skills)	Psychological capability, that is, procedural gardening knowledge Physical opportunity, that is, not having the time, funds or appropriate space/location to grow	Demonstration of the behaviour Instruction on how to perform a behaviour Behavioural practice/rehearsal
Modelling (Showing examples of the behaviour for people to imitate)	Social opportunity, that is, sense of community, prior experience learning from someone Automatic motivation, that is, rewarding feelings associated with gardening, for example, enjoyment and relaxation	Demonstration of the behaviour Social comparison Information about emotional consequences
Enablement (Providing support to change behaviour in ways not covered by other intervention functions, for example, through encouragement, moral support)	Psychological capability, that is, knowledge of front gardening benefits, procedural gardening knowledge, plant knowledge Social opportunity, that is, sense of community, prior experience learning from someone Physical opportunity, that is, not having the time, funds or appropriate space/location to grow. Having plants handed down by previous tenants/homeowners	Social support (unspecified) Social support (practical) Adding objects to the environment Restructuring the physical environment Social support (emotional) Restructuring the social environment

Similarly, potential implementation options were generated and evaluated against APEASE by the lead author (ALA), and reviewed independently by the co-authors (RF and NM). No major discrepancies were noted and minor discrepancies were discussed until resolved. For pragmatic reasons of protecting stakeholders’ time, a total of 12 intervention implementation options, balanced across the six selected intervention types were selected (the full list is in Table 6). The implementation options were refined and revised based on feedback from stakeholders (described in the subsequent section), and the final list of recommended interventions was generated.

**Table 6.** Intervention recommendations alongside their academic expert evaluations and consolidated stakeholder feedback

BCW intervention type	Intervention implementation options	APEASE evaluations of implementation options based on expert academic evaluations	Stakeholder evaluations of intervention implementation options (general public and local councillor)	Final decision on this intervention implementation option
Education (Increasing knowledge or understanding)	Creation of educational materials providing information to novice gardeners, for example, clear instruction that matches plants to different maintenance requirements, budget needs and garden specifications (space, location, size, condition). This educational strategy focusses on more specific practical/procedural awareness raising.	Considered *potentially* affordable, practical, *potentially* acceptable, should have limited side effects, and should not create significant issues of equity if tailored/targeted appropriately. Unlikely to be effective as a stand-alone strategy and best combined with other approaches. Knowledge/awareness is a necessary but insufficient driver of behaviour change.	The consensus was that specific education was a good idea because the garden can be an intimidating space for inexperienced gardeners. However, this information needs to consider the whole behavioural ‘journey’ by starting with how to clear/prepare a garden and providing guidance on how to maintain plants after they have been planted. Accessibility of informational materials will be important (e.g., accessible language, formats and visuals). Other important areas to provide specific information on included: wildlife (bees, birds, insects) and allergies (e.g., latex as some plants produce latex) to minimise potential issues relating to health and invasive species that might deter people.	This is a necessary but insufficient behaviour change strategy to promote front gardening. Ensuring that information is accessible and targeted towards different demographics and skill-levels will be important. Most likely to be effective in combination with other types of practical/social support to front garden.
	Production of educational material on the health, social and environmental benefits of growing in front gardens to ‘raise the profile’ of this behaviour. Content can be created for different multimedia formats (text, visual, audio). This educational strategy focusses on more generalist and motivational awareness raising.	Considered *potentially* affordable, practical, *potentially* acceptable, should have limited side effects, and should not create significant issues of equity if tailored/targeted appropriately. Unlikely to be effective as a stand-alone strategy and best combined with other approaches. This approach should be applied in conjunction with more specific practical capacity-building and persuasive approaches. These types of strategies can also be more difficult to evaluate in terms of their direct impact on any behaviour change.	The consensus was that this strategy might be effective but as it is very broad and general it would likely be effective more in terms of public consciousness raising and a slower, long-term cultural change. It was also deemed important to raise awareness of gardening responsibly by raising awareness of the potential impacts of gardening on neighbours, for example, noise from leaf blowers or smoke from controlled burns.	Unlikely to be effective on its own to promote front gardening specifically. This type of strategy would be best implemented alongside other specific educational and practical/social support strategies for front gardening.
Persuasion(Getting people to change behaviour by generating ‘cognitive dissonance’ – an uncomfortable state of having contradictory beliefs, thoughts or values towards something)	Production of content for media campaigns that raise the profile of and increase attractiveness of front gardening by depicting it to be a desirable behaviour that pro-social, pro-health and/or sustainability-conscious citizens do. Examples include ‘green gifting’. This could involve demonstrating plants suitable for front gardens as good housewarming, wedding, anniversary, memorial or birthday gifts. Other examples include linking front gardening with ‘self-care’. This could involve collection of short stories about peoples’ positive transformation experiences through enacting the target behaviour.	Considered *potentially* affordable depending on campaign budget, *potentially* practical, for example, could crowdsource material and leverage online content creators and *potentially* acceptable. To minimise negative side effects, it is important to avoid a shame-based marketing approach. There are potential issues with equity if the issue was too commercially based and driven by profit. These types of strategies could be adapted depending on the desired scope, for example, national vs. local. These types of strategies can be more difficult to evaluate in terms of their direct impact on any behaviour change.	The consensus was that while green gifting might work for some demographics, it is unlikely to be desirable for everyone as people may have other priorities. Stakeholders generally found such social marketing strategies problematic as they were seen as appropriating, co-opting and commercialising something that ought not to be, that is, self-care and environmental preservation. This could also add undue pressure on people to conform to standards and expectations that they might not be able to meet contributing to stress.	While such strategies could potentially be effective, they were not deemed acceptable by stakeholders due to the potential exploitation of citizens’ insecurities. This could cause harm to people’s well-being by making them feel ‘less than’.
	Sending of motivational letters from the local council/community groups to persuade residents to grow in front gardens. The letter could provide information about the behaviour of others within the borough or another nearby borough (motivation through descriptive social norms). Ideally the letter would also clearly signpost to resources that can support residents in engaging in the target behaviour, for example, informational websites, nearest garden centres and any funds they can access to support with associated costs, etc.	Considered *potentially* affordable and *potentially* practical. Such an approach may require some kind of initial scoping/investment of time and funds to identify the comparison for baseline or collect the data to include in the letter. Considered *potentially* acceptable; people may have issue what they perceive to be motivational communication, for example, ‘junk mail’. Issues relating to side effects could include excess generation of paper waste. To minimise issues with equity, would need to think about language and translation depending on the communities in the area. This, in turn, has implications for practicality and affordability. Social comparison (the type of strategy that this is) is likely to be effective but this is dependent on people reading and engaging with the letter and not putting it straight into their bins.	Motivational messaging/letters may have better engagement from local trusted community groups such as schools or faith-based organisations, rather than local governing authorities. The tone would also need to be important to avoid coming across as patronising. The responsibility of the local council was seen to be more for providing practical and logistical support and services, for instance, in the form of efficient and reliable garden waste collection services to support residents. This would likely be a much more effective use of local authority resources.	Such strategies are only likely to be effective if engagement is high – this will depend on who the communication is coming from and the tone of delivery. Even then, it is unlikely to be effective without combination with other specific educational and practical/social support strategies. Overall, this was not an effective strategy as people are already bombarded with written communications in their daily lives.
Incentivisation(Changing the attractiveness of a behaviour by creating the expectation of reward)	Creation of competitions within local boroughs and communities where there are prizes for the best front gardens/streets. There could be different prizes, for example, for gardens of different shapes, sizes, conditions, plant varieties, etc.	Considered *potentially* affordable, *potentially* acceptable, should have limited side effects, and should not create significant issues of equity as there would be different winner categories. When evaluating, any comparison boundaries between groups should reflect place identities. There are potential issues with practicality and effectiveness in the first instance. You would likely need a few enthusiasts to begin with and let it slowly build over time. This type of strategy would be part of a slower, long-term cultural change.	Some people felt that competitions could be effective and acceptable if they were centred around on community-building and inclusivity. Otherwise, something like this could raise tensions. Issues were raised in terms of potentially excluding community members who do not have the time, resources or front gardens to participate.	Likely to be effective in a local capacity. Centring the competitions around community-building and inclusivity will be important to promote social cohesion.
	Provisions of free plants to residents. This could be done by local garden centres, community gardens, gardening groups.	Considered *potentially* affordable, practical, acceptable, should have limited negative side effects and should not create significant issues of equity. However, difficult to ascertain effectiveness as difficult to know whether they will get put in front gardens. Nonetheless, promoting gardening activity is likely to have a spill over effect into front gardening,	The consensus was that it would always be a good idea to make plants more accessible but there is little way to ensure the plants provided end up in the front garden (vs. the back garden or inside the house). It was also suggested that giving away soil, stones and gardening equipment freely would also be helpful.	Likely to be very effective. To increase likelihood that plants are placed in front gardens, only give away items (plants, pots, etc.) most compatible for front gardens.
	Get people with excess plants (residents or local businesses) to give extra plants (or those that will die/end up as waste) away via apps or social media, for example, local WhatsApp groups, Too Good To Go or Olio. Such processes and platforms are already associated with the ‘waste-reduction’, pro-environmental movement; this can be leveraged to incentivise people to purchase cheaper plants or pick up free ones for their front gardens.	Considered affordable, acceptable and practical as piggybacking onto existing infrastructure. Potentially effective as it may require some time for something like this to become mainstream. Minimal issues with side effects anticipated. Minimal issues with equity except people would need to have access to Internet and smartphones.	The consensus was that sharing / hire networks would be a good way to make plants more accessible to community members. However, it is not just plants / seedlings that people need, it is also soil, stones and general gardening equipment and so these networks would ideally also freely or affordably share these too.	Likely to be effective and have positive side effects of increasing social cohesion. Unclear how effective it would be for growing in private front gardens specifically vs. gardening more generally.
Training(Increasing psychological or physical skills)	Develop events and workshops where people can learn and enhance or practice front gardening skills. These could be run by schools, city farms, parks, zoos, community gardens, etc. Events can be tailored to meet the abilities, priorities and garden conditions of different groups, for example, school-aged children, adolescents, young adults, queer groups, people with disabilities, older adults, etc.	Considered *potentially* affordable and *potentially* practical. This strategy is easily adapted to different resources; one will just need to source experts to facilitate training. Minimal issues with negative side effects anticipated. Minimal issues with equity if a tailored/inclusive approach is taken. *Potentially* effective but this may be limited to smaller groups of people. Also run the risk of ‘preaching to the converted’. Could be done in collaboration with other organisations, for example, city farms, schools, new parent groups, etc.	There were generally positive feelings about training and workshops because they can build skills while building community. It was deemed important for the programmes to run for long enough so that people have enough time to practice skills and build confidence before they implement in their own gardens. Accessibility was deemed important, as was remembering that gardening is not always therapeutic for everyone owing to differences in abilities, allergies or risk of sunburn.	Likely to be effective and have positive side effects of increasing social cohesion. Unclear how effective it would be for growing in private front gardens specifically vs. gardening more generally. For high engagement, ensure that training is accessible and targeted towards different demographics and skill-levels.
Modelling(Showing examples of the behaviour for people to imitate)	Production of content for media campaigns in the form of examples of ‘desirable’ front gardens (of all different sizes/conditions) for people to aspire to. These could include ‘before and after’ photos of gardens that have been transformed to be ‘greener’, more aesthetically appealing or spaces that have been rewilded. Examples should be matched towards different garden types, sizes, conditions, maintenance needs and budgets. This strategy is focussed on modelling aspirational goals.	Considered *potentially* affordable, practical as easily adaptable for different social media platforms (e.g., Pinterest, TikTok, YouTube, Instagram), acceptable, should have limited negative side effects and should not create significant issues of equity. Considered *potentially* effective depending on how targeted the campaign, relatable the content creators are and the mode of delivery. This strategy is difficult to evaluate in terms of its direct impact on any behaviour change. It may also need to be matched with practical process-related education/support as people may know what their end goal is (i.e., a desirable garden) but not be so sure how to get there.	The consensus was that this is a good strategy and likely to be effective if the example gardens resonate with people’s desires and is considerate of and tailored towards people’s needs, priorities and resources. There were questions around equity, for example, what is a reasonable thing for people to feel like they can aspire to? People have different preferences and desires when it comes to their gardens. Some people like neat lawns while others like bees and a jungle of wildflowers. It was suggested that this this diversity in preferences would need to be reflected in campaigns too. It was unclear how one might assess for any direct relationships between such strategies and any changes in behaviour.	Unlikely to be effective as a stand-alone strategy. More likely to be effective in combination with other types of specifical, tailored educational and practical/social support strategies to front garden. So as not to cause unintended side effects, it is important to make sure such strategies were as inclusive and diverse as possible, otherwise it could be seen to be exploiting people’s insecurities. Such strategies would need to be considerate of different values, preferences and lived experiences.
	Production of content for media campaigns in the form of ‘relatable’ people sharing their experiences of front gardening to persuade others (‘if I can do it, so can you’). The idea would be to provide moral support by building confidence and increasing a sense of self-efficacy. The diversity of the experiences collected should reflect the diversity of the people being targeted. This strategy is focussed on modelling a desirable feeling, that is, feeling represented and confident in yourself.	Considered *potentially* affordable, practical as easily adaptable for different social media platforms (e.g., Pinterest, TikTok, YouTube, Instagram), acceptable, should have limited negative side effects and should not create significant issues of equity. Considered *potentially* effective depending on how targeted the campaign, relatable the content creators are and the mode of delivery. This strategy is difficult to evaluate in terms of its direct impact on any behaviour change.	The consensus was that this is generally a good idea and likely to be effective if executed appropriately. Garden buddies and ambassadors were suggested as a potential way to passively model and encourage behaviour. Showing people how to break gardening down into manageable chunks was deemed potentially helpful, for example, focussing not on the whole garden but starting with a small area or mini project such as a few planters, pond or birdfeeder. It was unclear how one might assess for any direct relationships between such strategies and any changes in behaviour.	Likely to be effective as the ‘buddy’ could ensure any growing was in the front garden. This type of strategy would have additional benefits in terms of the socialising and community building. Could be potentially resource-intensive sourcing local, experienced gardeners to volunteer their time.
Enablement(Providing support to change behaviour in ways not covered by other intervention functions, for example, through encouragement, moral support)	Creation of ‘front garden growing’ starter kits available for purchase from garden centres or local gardening enthusiasts. They should be tailored to meet the needs of different budgets, maintenance requirements and garden conditions. This is best paired with good educational content providing procedural knowledge of what the plants are and how to care for and maintain them.	Considered *potentially* affordable depending on how they are priced. Considered acceptable and effective as people are unlikely to make the financial investment and then not put the plants in their front gardens. Considered practical as this intervention substantially reduces the time and effort barriers to adopting a new behaviour. Limited issues with negative side effects anticipated. To minimise issues with equity, need to make sure they are accessible, for example, have home-delivery options	The consensus was that this is a good idea but would be even better if there was a way to do this freely, for example, donated plants and garden equipment. Other locations where such kits could be distributed included community food banks and charity shops.	Likely to be very effective as it greatly minimises the intimal time, financial and effort costs, especially if there was a way to do this entirely free of charge.
	Creation of ‘in real life’ or social media networks and groups aimed specifically at promoting knowledge exchange, sharing gardening tools, donating seedings and plants and fostering a sense of community building. These can also be good places to advertise events and workshops.	Considered affordable, acceptable and practical. Existing networks may already be in place, and they could be leveraged. New groups could be created through there are potential resource costs associated with initial set-up. This has implications for effectiveness as it may take some time for such groups to be effective until there is more engagement with them. Also, unlikely to be very effective as a stand-alone strategy. This intervention strategy is best combined with others and seen as core foundational component as it provides a building block and baseline structure for advertising, awareness raising, knowledge sharing, networking and connecting people. Limited issues with negative side effects anticipated, though this depends on group dynamics. Limited issues with equity anticipated, though people will need to have access to smartphones, computers and Internet if these groups are online.	The consensus was that this could potentially be effective if groups were effectively moderated and there was not information overload. Protecting the safety and security of group members was deemed important, for example, if people are exchanging addresses to share plants/gardening equipment.	Unlikely to be effective as a stand-alone strategy but would work in combination with other strategies. There are potential issues with information overload if the network is digital, it would need to be run effectively for there to be high engagement. Nonetheless, having a foundational network of some sorts will be important as it can serve as an intervention platform and networking and knowledge sharing space.

#### Stakeholder consultation

The final round of review for the interventions was with two groups of stakeholders: members of the general public with access to a front garden, and local councillors involved in sustainability initiatives. Stakeholders were consulted for their feedback on the practicability, relevance, utility and acceptability of the proposed interventions. The consultations also provided an opportunity for feedback on the behavioural map, as a valuable resource in understanding the wider behavioural system of front gardening.

The members of the public were consulted via a 1.5-h virtual workshop. Workshop participants (n = 7) consisted of working-age adults and were recruited via a panel of individuals registered to support health research in the UK. The key inclusion criterion was an interest in the topic of front gardening in the individual’s response to the recruitment flyer. While limited by the range of people who responded to the study advert, we tried to select participants to provide maximum diversity concerning age, ethnicity, gender, living with disability; location (urban/rural); housing tenure (tenant/owner); and experience of front gardening (experienced/novice) (see Table 7 for demographics). The selected stakeholders were sent a document with the interventions to review ahead of the workshop and all workshop attendees were encouraged to contribute. Stakeholders were reimbursed for their time with a £50 voucher.

**Table 7.** General public stakeholder characteristics

Stakeholder	Gender	Ethnicity	UK region	Age
1	Female	White British	Bath, Somerset	51
2	Female	White British	Norfolk, East Anglia	48
3	Female	British Pakistani	Bradford, West Yorkshire	53
4	Female	British Indian	London Borough of Tower Hamlets	48
5	Female	British Indian	London Borough of Hillingdon	44
6	Female	White American	London Borough of Barnet	65
7	Male	White British	London Borough of Richmond	65

On the basis that councillors involved in local government in the UK have experience of seeking to change local residents’ behaviour on a variety of issues, local councillor stakeholders were consulted. Local councillors were recruited via one author’s (NM) links to local low-carbon initiatives in Hertfordshire. An email was sent out advertising the study and asking for feedback on the intervention recommendations. Local councillors who expressed interest were sent a document with the interventions to review. We received feedback from n = 4 local councillors. Two councillors served on a local planning subcommittee of a Parish Council. Two were District Councillors and members of the Green Party who provided a combined response.

Based on the analytic steps and stakeholder feedback outlined above, we developed a final shortlist of promising interventions, including what further considerations would be needed before evaluation or implementation.

**Figure 4:**
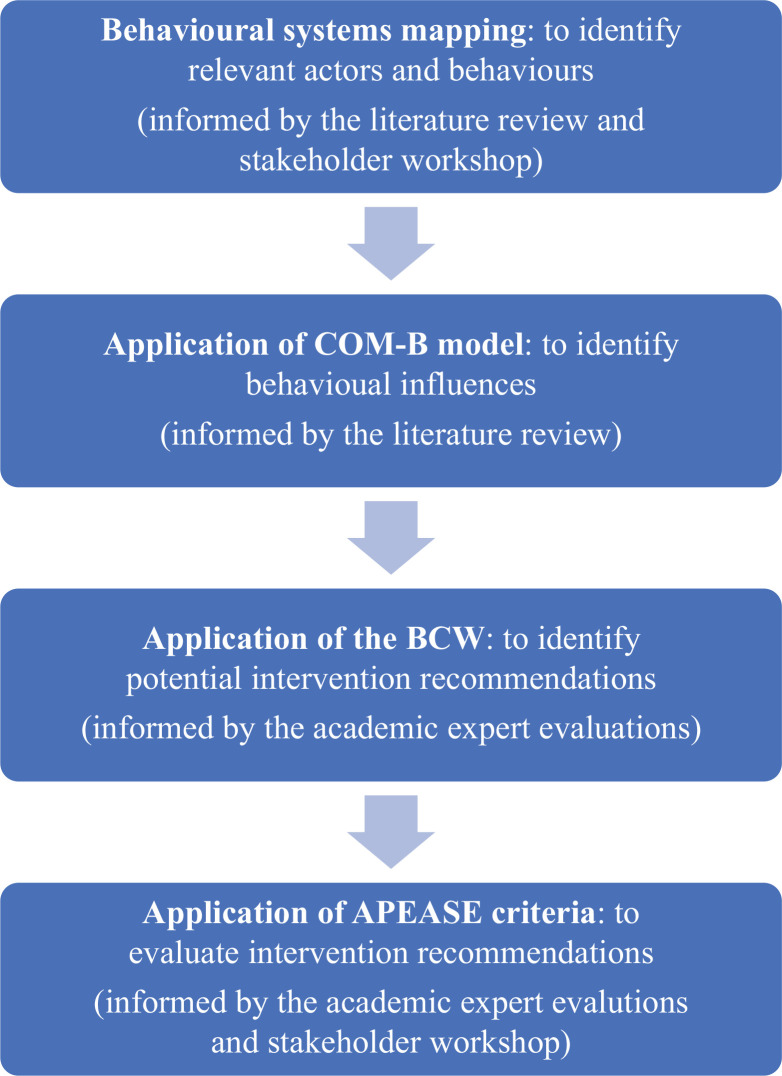
A visual summary of the intervention recommendation development process informed by the overarching BCW method.

Figure 4 provides a visualised summary of the intervention development process.

## Results

### The behavioural system

**Figure 5:**
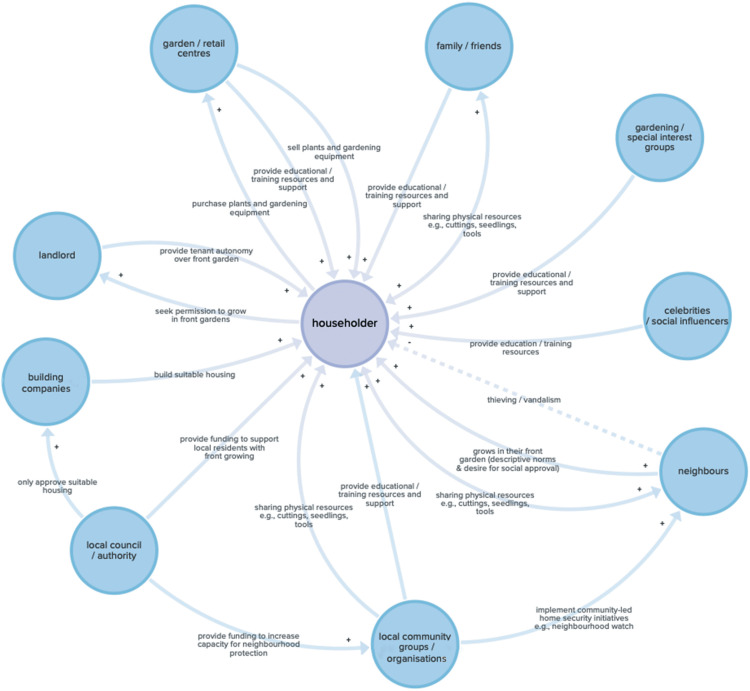
A behavioural systems map depicting the key actors and behaviours involved in front gardening amongst UK householders. Note: + = an increase in this behaviour makes front gardening *more* likely, − = an increase in this behaviour makes front gardening *less* likely. A double arrow with a +/− on both sides indicates that the direction of influence goes both ways.

Figure 5 illustrates the front gardening behavioural systems map, consisting of high-level actors, that is, broad groups of people relevant to front gardening, connected via behaviours that either increase or inhibit front gardening.

The stakeholder consultations identified the actors considered most important for influencing householder front gardening as: local community groups/organisations, neighbours, family (especially children in the household who might learn about gardening at school) and friends, via influencing the skills, perceptions and behaviours of households.

### Intervention recommendations

Valuable insights were gathered from the stakeholder consultations, supplementing the academic expert intervention evaluations. Table 6 highlights the final set of intervention recommendations alongside the academic expert evaluations and consolidated stakeholder feedback. Both the public stakeholders and local councillors felt that the behavioural systems map and proposed set of interventions were comprehensive and covered the wide range of potential barriers to front gardening.

The public stakeholder workshop revealed that educational and supportive (both practical and social) strategies were preferred to persuasive or aspirational ones. Public stakeholders felt that gardening was a personal and cultural practice and were less comfortable with the idea of persuasive strategies aimed at motivating householders to meet certain ideals, particularly if it was coming from businesses (whose primary aim was viewed as generating profit) or local governing authorities (whose priorities were viewed as better placed elsewhere). The responsibility of local authorities was viewed as providing practical support and better public services to citizens, not telling them how they should garden via letters, which was viewed as potentially patronising and a waste of council resources. The council was deemed better suited to help in the areas of funding, improvements to local parking and better waste collection services (for garden waste and other types of waste more generally) to enable front gardening. This sentiment was echoed by the local councillors who also agreed that letters or other types of communications from the local authority would not be effective.

Throughout the public stakeholder workshop discussion, there was an emphasis on inclusivity and accessibility; so as not to widen existing disparities in society or cause further harm to health and well-being, it was advised that any behaviour change strategy should be sensitive of the diverse range of values, abilities, preferences and lived experiences of the UK public. This was particularly the case for interventions including persuasion and modelling. It was also highlighted that some strategies may not be specific to front gardening behaviour (vs. all gardening behaviour). Although encouraging any gardening behaviour could be viewed as a positive result, specificity is needed to provide the unique benefits associated with greener front gardens.

The importance of engaging young people (e.g., children and teenagers) within households was highlighted by both public stakeholders and local councillors. Furthermore, to make front gardening easier and more accessible, both public stakeholders and local councillors felt that practical and social support interventions should not only focus on providing plants (e.g., seedlings) but on making soil, stones, pots and gardening equipment, for example, watering cans, more accessible. Public stakeholders also mentioned additional challenges faced by householders who did not own their homes. Council tenants and renters often face restrictions in what they can do to their gardens. Having to seek permission from landlords or housing authorities adds a layer of bureaucracy that can hamper gardening efforts.

The final recommendations are that information strategies, while necessary, are unlikely to be sufficient drivers of behaviour change. Ensuring that any information provision is accessible and tailored towards different demographics, budgets and skill-levels will be important. Persuasive strategies are only likely to be effective and acceptable if they are not perceived to be coercive or exploitative and thus potentially harmful to householders’ mental health. While lowering initial time, effort and financial costs will be effective for onboarding householders (e.g., free plants and equipment), implementing strategies that build nature- and community-connectedness and promote social cohesion are likely to be most acceptable by intervention recipients and ensure behaviour change is maintained in the long-term.

## Discussion

This study aimed to determine a set of behaviour change intervention recommendations promoting front gardening amongst UK householders. A secondary aim of this study was to develop these intervention recommendations by systematically applying an established behaviour change intervention development framework – the BCW. Our method involved a rigorous and structured design process built on a foundation of behaviour change theory and peer reviewed scientific evidence. Academic expert evaluations and UK local councillor and public stakeholder feedback also informed the intervention recommendation development process. The findings aligned with previous research, which is sparse as mentioned earlier. Given the paucity of empirical evidence focussed specifically on front gardening promotion, contextualising the findings of this study within the wider evidence-base relied on extrapolating from the findings of related behaviours and contexts.

A range of intervention approaches were deemed potentially implementable. Those with the highest promise for the target behaviour of gardening were found to be capacity-building such as educational (e.g., increasing knowledge, awareness and skills) and supportive (practical, moral and social) strategies (e.g., community workshops, building social networks, sharing initiatives, distribution of free resources). This was over and above persuasive or aspirational strategies, which could be potentially unethical and coercive (e.g., aspirational social media campaigns depicting ‘desirable gardens’). Incentivisation strategies were also deemed acceptable as long as they focussed on the positive (e.g., ‘freebies’) as opposed to creating an expectation of punishment or loss of esteem (e.g., creating competition). This aligns with prior behavioural research indicating that shame-based (e.g., creating stigma) [45] or fear-based approaches [46] raise significant questions around long-term effectiveness, negative side effects and equity of interventions. Prior studies also indicate the importance of ongoing resources and training to maintain long-term desired outcomes in community health-based interventions [47].

The stakeholder consultations revealed that householders’ front gardening behaviour can be understood as a collection of related sub-behaviours including clearing out an area of land, sourcing plants and garden equipment, maintaining the garden, to disposal of garden waste, each with their own set of barriers and enablers. While lowering initial time, effort and financial costs are most likely to be effective for engaging householders who do not currently garden (e.g., free plants and equipment), implementing strategies that build nature- and community-connectedness and promote social cohesion is likely to be the most effective, equitable and sustainable in the long term. These findings also highlight how behavioural change is a process, rather than having an ‘on/off’ switch. It is well documented in the literature that behaviour change interventions can be effective in achieving temporary behaviour change but less effective at behaviour change maintenance [48]. A review of behaviour change maintenance theories showed that self-regulation, personal and social resources, habits, and environmental and social influences were effective at maintaining behaviour [48]. Therefore, some of the intervention recommendations will likely be more effective at supporting behaviour change and/or maintenance depending on where someone is in their gardening ‘journey’.

In earlier work on engaging non-gardeners in wildlife gardening programmes, the strategies that were most successful at recruiting previously unengaged members were providing site assessments and native plants or vouchers to members [49]. Evidence also shows that people are more likely to maintain a household garden if they also demonstrate high community engagement, for example, through participation in alternative and local food systems [50]. In further support of our recommendations, intervention programmes that strengthen nature-connectedness and facilitate communication about wildlife gardening (i.e., building knowledge) between friends and family (i.e., building community) have been recommended by prior researchers examining the factors influencing engagement in gardening practices that support biodiversity [51].

### Theoretical and practical implications

A theoretical contribution of this study is the documentation of a systematic intervention development framework application process within a novel implementation context (i.e., front gardening). There is a paucity of intervention development studies in academic journals [52]. When intervention development studies are published, they are usually included as part of a feasibility or pilot study [53]. Publishing documentation of the intervention development process as standalone papers, and in line with established frameworks and guidance (e.g., the BCW), allows for a more systematic and transparent approach to intervention development. This, in turn, enhances the quality of interventions and improves learning about intervention development research and practice thereby advancing applied behavioural science.

A practical contribution is in the generation of a series of recommendations for interventions. Our structured approach and stakeholder feedback indicate that knowledge-based campaigns on front gardening, such as social media campaigns, are likely to be insufficient to change behaviour without being paired with other local interventions. It therefore strongly supports the need for local community-based approaches for encouraging front gardening. Whilst this may be challenging within the current climate of funding difficulties for local councils, our work explored a range of options to encourage exchanges of plants and materials between residents or from local businesses, and these options were seen as affordable, practical and feasible (although somewhat lacking in specificity to front gardens).

### Strengths, limitations and future research

The engagement of stakeholders, that is, ‘experts by experience’ [54], was a key strength of this study. It is recognised that involving people who are representative of those who might deliver (e.g., local councillors) or receive (e.g., the general public) interventions enhances the likely quality, equity, relevance and long-term sustainability of interventions [55–57]. Stakeholders also often possess valuable insights into the specific needs, challenges and preferences of the target population. Their input therefore can ensure that the intervention is tailored to address these factors, making it more relevant and effective. Stakeholders can also provide practical insights into the feasibility of implementing the intervention. As they are ‘on the ground’, they can identify potential barriers, resource constraints and operational challenges, helping to refine the design for better practicality – indeed, these are all insights gained during the public and local councillor stakeholder consultations conducted for this study.

Another key strength of this study was utilisation of the BCW to guide intervention development. While there are other intervention development frameworks, for example, intervention mapping [58], some of the key benefits of the BCW include its flexibility. Intervention mapping focusses mostly on health promotions and health communications while the BCW is adaptable (and has been adapted) to various contexts and behaviours, as demonstrated by this study. It is not limited to a specific behaviour, context or population, making it versatile for addressing a wide range of challenges. The systematic approach based on BCW framework also enabled a limited and defensible set of appropriate interventions that could be proposed for discussion with stakeholders. This was not only practical in terms of stakeholder time but it also helped assure stakeholders of a rigorous intervention development method based on previous research and theory.

The application of behavioural systems mapping to visualise the key actors and relationships involved in front gardening was another strength of this study. Not only is the map itself a novel contribution, it also served as a useful communication tool during the stakeholder consultations. By discussing the map, we were able to elicit stakeholders’ insights and experiences related to different elements of the front gardening ‘system’. This helped to highlight key leverage points and populations (e.g., schools and children) where changes could have a significant impact on the system.

A further strength of this study is the expansion of the BCW to a novel implementation context – front gardening. As evidenced by the number of societal problems that could be improved by behaviour change, applications of behavioural science are required in many areas beyond healthcare which is where the framework has predominantly been applied. Advancing behavioural science requires documentation of the application of intervention development frameworks to a wide range of behavioural domains including environmentally significant behaviours. Having a diversity of behavioural case studies to draw upon within the interdisciplinary, peer-reviewed evidence-base is useful in illustrating the benefits of the BCW approach and disseminating learning across disciplinary boundaries; for instance, in this case, between behavioural/implementation science and horticultural science.

Limitations of this study include the relatively narrow demographic of the public stakeholders consulted. For example, schools and children were highlighted as important actors while landlords and celebrities/social influencers were not. Similarly, social media campaigns were not deemed to be a promising behaviour change strategy. These perceptions are likely to have reflected the lived experiences of the stakeholders. For other segments of the UK public, with other lived experiences, for example, young adults who often rent, are more transient, ‘digital natives’ and often child-free, it is plausible that other actors may deem different intervention approaches more influential for enabling front gardening. Further, over half of the stakeholders were London residents – their views are unlikely representative of all of the UK.

Another potential limitation is the focus on recommending interventions for local community groups. We recognise that there are limitations to what a local community can achieve without higher-level policy or structural change in related areas, for example, waste collection, housing and car parking. For instance, barriers to easy on-street parking are likely to lead to people paving over their front gardens to make space for parking [16]. Our stakeholder consultations also revealed that housing tenants could face restrictions from landlords or housing associations on what they could plant in their gardens. The barriers to home ownership faced by many young adults acts as an additional barrier to front gardening – householders are unlikely to want to invest time, effort and financial resources in gardens that are not ‘theirs’ [20]. The stakeholder consultations also showed that better services for collecting garden waste would also likely enable people to garden more. While making intervention recommendations for housing, waste collection or parking policy was beyond the scope of this study, we recognise that efforts to promote front gardening would benefit from concurrently considering improvements to policies, regulations, infrastructure and public services in these areas.

Further limitations include the development of intervention recommendations specific to the UK context. While recognising that this may limit the transferability of our study’s findings and intervention recommendations, the value of this study lies in the demonstration of a method that is general and could easily be applied by a local authority or community group that wants to develop interventions promoting a gardening behaviour in their own context. In any case, behaviour is context-specific; behaviour change strategies are more likely to be effective when they are sensitive to their unique implementation context. Our step-by-step documentation of the intervention recommendation development process has demonstrated a transferrable methodology and created a series of useful research materials (i.e., tables) which can be used as guiding templates by other researchers and practitioners.

The next step for our intervention recommendations is implementation and evaluation, which, in turn, has implications for policies and practices sustaining environmental and community health. Future research may also wish to investigate the potential for national policy or local parking and garden waste collection interventions to increase front gardening amongst UK householders.

## Conclusions

Using structured behaviour change frameworks, such as the BCW and behavioural systems mapping, supported the development of intervention recommendations aimed at promoting planting in front gardens amongst UK householders. The behavioural systems map enabled conceptualisation of the issue and a useful communication tool, while the BCW enabled a limited and defensible set of appropriate interventions that could be proposed for discussion with stakeholders. This was not only practical in terms of stakeholder time but it also helped assure stakeholders of a rigorous intervention development method based on previous research and theory. These factors helped maximise stakeholder engagement and input, ensuring that the final behavioural systems map and intervention recommendations were as comprehensive and accurate as possible. We recommend that other researchers use a similar approach to intervention development when considering householder behaviour change.

Future research should implement and evaluate educational and supportive strategies such as community workshops, sharing initiatives and distribution of free resources. To ensure maximum effectiveness they should be tailored to the diverse skills, budget, maintenance needs and preferences of UK householders. It will be useful to understand the real-world effectiveness and short- and long-term impacts of such initiatives on behaviour change and levels of garden greenery.

## Data Availability

Data sharing not applicable to this article as no datasets were generated or analysed during the current study.

## Appendix

**Appendix A.** Intervention types appropriate for targeting underlying behavioural influences

COM-B	Intervention type	Definition	APEASE	Included/exclude from next stage
Psychological capability (i.e., knowledge of front gardening benefits, procedural gardening knowledge, plant knowledge)	Education	Increasing knowledge or understanding	Considered affordable, practical, *potentially* effective, *potentially* acceptable, should have limited side effects, and should not create significant issues of equity if targeted/tailored appropriately.	Include
	Training	Imparting skills	Considered *potentially* affordable, *potentially* practical, *potentially* effective, *potentially* acceptable, should have limited side effects, and should not create significant issues of equity if targeted/tailored appropriately.	Include
	Enablement	Providing support to improve ability to change in a variety of ways not covered by other intervention types, for example, through encouragement, moral support	Considered *potentially* affordable, *potentially* practical, *potentially* effective, *potentially* acceptable, should have limited side effects, and should not create significant issues of equity if targeted/tailored appropriately.	Include
Social opportunity (i.e., sense of community)	Restriction	Constraining behaviour by setting rules	Considered *potentially* affordable, but not practical as it would require enforcement (which in turn may reduce affordability), *potentially* effective, but not acceptable. Could have potential negative side effects and could create issues of equity.	Exclude
	Environmental restructuring	Changing the physical or social context	While likely to be effective, acceptable and have positive implications for equity, this is not considered affordable or practical, for example, cannot change the location of the home. Improving the socioeconomics of the neighbourhood is complex and beyond the scope of a research study. Walling off front gardens could provide increased protection of the garden but could lead to unintended consequences such as increasing isolation and making the neighbourhood appear more unsafe.	Exclude
	Modelling	Providing an example for people to aspire to or imitate	Considered *potentially* affordable, practical, *potentially* effective, *potentially* acceptable, should have limited side effects, and should not create significant issues of equity.	Include
	Enablement	Providing support to improve ability to change in a variety of ways not covered by other intervention types, for example, through encouragement, moral support	Considered *potentially* affordable, *potentially* practical, *potentially* effective, *potentially* acceptable, should have limited side effects, and should not create significant issues of equity.	Include
Physical opportunity (i.e., not having the time, funds or appropriate space/location to grow. Having plants handed down through previous tenants/homeowners)	Training	Imparting skills	Considered *potentially* affordable, practical, *potentially* effective, *potentially* acceptable, should have limited side effects, and should not create significant issues of equity if targeted/tailored appropriately.	Include
	Restriction	Constraining behaviour by setting rules	Not considered practical to the physical opportunity related behavioural influences identified, for example, cannot set rules to alter size/location of garden or provide people with more time and money.	Exclude
	Environmental restructuring	Changing the physical or social context	Not considered practical to the physical opportunity related behavioural influences identified, for example, cannot change the size/location of gardens.	Exclude
	Enablement	Providing support to improve ability to change in a variety of ways not covered by other intervention types, for example, through encouragement, moral support	Considered *potentially* affordable, practical, *potentially* effective, *potentially* acceptable, should have limited side effects, and should not create significant issues of equity if targeted/tailored appropriately.	Include
Automatic motivation (i.e., rewarding feelings associated with gardening, e.g., enjoyment and relaxation)	Persuasion	Changing the way people feel about a behaviour by generating cognitive dissonance and showing how changing behaviour can reduce it	Considered *potentially* affordable, practical, *potentially* effective, *potentially* acceptable, should have limited side effects, and should not create significant issues of equity.	Include
	Incentivisation	Changing the attractiveness of a behaviour by creating the expectation of a desired outcome or avoidance of an undesired one	Not considered practical to the automatic motivation related behavioural influences identified.	Exclude
	Coercion	Changing the attractiveness of a behaviour by creating the expectation of an undesired outcome or denial of a desired one	Not considered practical to the automatic motivation related behavioural influences identified.	Exclude
	Training	Imparting skills	Not considered practical to the automatic motivation related behavioural influences identified.	Exclude
	Environmental restructuring	Changing the physical or social context	Not considered practical to the automatic motivation related behavioural influences identified.	Exclude
	Modelling	Providing an example for people to aspire to or imitate	Considered *potentially* affordable, practical, *potentially* effective, *potentially* acceptable, should have limited side effects, and should not create significant issues of equity.	Include
	Enablement	Providing support to improve ability to change in a variety of ways not covered by other intervention types, for example, through encouragement, moral support	Not considered practical to the automatic motivation related behavioural influences identified.	Exclude
Reflective motivation (i.e., gardening as form of creativity/self-expression and identity, e.g., pride, connectedness, responsibility, civic duty. High self-efficacy and confidence in capabilities. Competing priorities and absence of motivation to front garden)	Education	Increasing knowledge or understanding	Not considered practical to the reflective motivation related behavioural influences identified.	Exclude
	Persuasion	Using communication to induce positive or negative feelings to stimulate action	Considered *potentially* affordable, practical, *potentially* effective, *potentially* acceptable, should have limited side effects, and should not create significant issues of equity if targeted/tailored appropriately.	Include
	Incentivisation	Changing the attractiveness of a behaviour by creating the expectation of a desired outcome or avoidance of an undesired one	Considered *potentially* affordable, practical, *potentially* effective, *potentially* acceptable, should have limited side effects, and should not create significant issues of equity if targeted/tailored appropriately.	Include
	Coercion	Changing the attractiveness of a behaviour by creating the expectation of an undesired outcome or denial of a desired one	Considered *potentially* affordable, *potentially* practical, *potentially* effective, but significant issues with acceptability and could have negative side effects and create issues of equity.	Exclude

**Appendix B.** Identification of potential BCTs that could be used in the intervention as recommended by the BCW guide

Intervention type selected	COM-B component targeted	Potential BCTs (most frequently used)	Potential BCTs (less frequently used)
Education	Psychological capability, that is, knowledge of front gardening benefits, plant knowledge	**Information about social and environmental consequences** **Information about health consequences** Feedback on behaviour Feedback on outcome(s) of behaviour Prompts/cues Self-monitoring of behaviour	Biofeedback Self-monitoring of outcome(s) of behaviour Cue signalling reward Satiation **Information about antecedents** Re-attribution **Information about emotional consequences** Information about others’ approval
Persuasion	Automatic motivation, that is, rewarding feelings associated with gardening, for example, enjoyment and relaxation Reflective motivation, that is, gardening as form of creativity/self-expression and identity, for example, pride, connectedness, responsibility, civic duty. High self-efficacy and confidence in capabilities	Credible source **Information about social and environmental consequences** **Information about health consequences** Feedback on behaviour Feedback on outcome(s) of behaviour	Biofeedback Re-attribution **Focus on past success** **Verbal persuasion about capability** Framing/reframing **Identity association with changed behaviour** **Identification of self as role model** **Information about emotional consequences** Salience of consequences **Information about others’ approval** **Social comparison**
Incentivisation	Reflective motivation, that is, competing priorities and lack of intention to front garden	Feedback on behaviour Feedback on outcome(s) of behaviour Monitoring of behaviour by others without evidence of feedback Monitoring of outcome of behaviour by others without evidence of feedback Self-monitoring of behaviour	Paradoxical instructions Biofeedback Self-monitoring of outcome(s) of behaviour Cue signalling rewards Remove aversive stimulus Reward approximation Rewarding completion Situation-specific reward Reward incompatible behaviour Reduce reward frequency Reward alternative behaviour Remove punishment Social reward Material reward Material reward (outcome) Self-reward Non-specific reward **Incentive** Behavioural contract Commitment Discrepancy between current behaviour and goal Imaginary reward
Training	Psychological capability, that is, procedural gardening knowledge Physical opportunity, that is, not having the time, funds or appropriate space / location to grow (*e.g., can reduce barriers by demonstrating low-cost, low-maintenance growing options for spaces of all shapes and sizes*)	**Demonstration of the behaviour** **Instruction on how to perform a behaviour** Feedback on the behaviour Feedback on outcome(s) of behaviour Self-monitoring of behaviour **Behavioural practice/rehearsal**	Biofeedback Self-monitoring of outcome(s) of behaviour Habit formation Habit reversal Grade tasks Behavioural experiments Mental rehearsal of successful performance Self-talk Self-reward
Modelling	Social opportunity, that is, sense of community Automatic motivation, that is, rewarding feelings associated with gardening, for example, enjoyment and relaxation	**Demonstration of the behaviour**	
Enablement	Psychological capability, that is, knowledge of front gardening benefits, procedural gardening knowledge, plant knowledge Social opportunity, that is, sense of community Physical opportunity, that is, not having the time, funds or appropriate space/location to grow. Having plants handed down by previous tenants/homeowners	**Social support (unspecified)** **Social support (practical)** Goal setting (behaviour) Goal setting (outcome) **Adding objects to the environment** Problem solving Action planning Self-monitoring of behaviour **Restructuring the physical environment** Review behaviour goal(s) Review outcome goal(s)	**Social support (emotional)** Reduce negative emotions Conserve mental resources Pharmacological support Self-monitoring of outcome(s) of behaviour Behaviour substitution Overcorrection Generalisation of a target behaviour Graded tasks Avoidance/reducing exposure to cues for the behaviour **Restructuring the social environment** Distraction Body changes Behavioural experiments Mental rehearsal of successful performance Focus on past success Self-talk Verbal persuasion about capability Self-reward Behavioural contract Commitment Discrepancy between current behaviour and goal Pros and cons Comparative imagining of future outcomes Valued self-identity Framing/reframing Incompatible beliefs Identity associated with changed behaviour Identification of self as role model Salience of consequences Monitoring of emotional consequences Anticipated regrets Imaginary punishment Imaginary reward Vicarious consequences

**Bold** = selected BCTs.
